# Similar But Different: Integrated Phylogenetic Analysis of Austrian and Swiss HIV-1 Sequences Reveal Differences in Transmission Patterns of the Local HIV-1 Epidemics

**DOI:** 10.1097/QAI.0000000000002949

**Published:** 2022-03-17

**Authors:** Katharina Kusejko, Nadine Tschumi, Sandra E. Chaudron, Huyen Nguyen, Manuel Battegay, Enos Bernasconi, Jürg Böni, Michael Huber, Alexandra Calmy, Matthias Cavassini, Alexander Egle, Katharina Grabmeier-Pfistershammer, Bernhard Haas, Hans Hirsch, Thomas Klimkait, Angela Öllinger, Matthieu Perreau, Alban Ramette, Baharak Babouee Flury, Mario Sarcletti, Alexandra Scherrer, Patrick Schmid, Sabine Yerly, Robert Zangerle, Huldrych F. Günthard, Roger D. Kouyos

**Affiliations:** a Division of Infectious Diseases and Hospital Epidemiology, University Hospital Zurich, Zurich, Switzerland;; b Institute of Medical Virology, University of Zurich, Zurich, Switzerland;; c Department of Medicine, Swiss Tropical and Public Health Institute, Basel, Switzerland;; d Department of Clinical Research, University of Basel, Basel, Switzerland;; e Division of Infectious Diseases and Hospital Epidemiology, University Hospital Basel, University of Basel, Basel, Switzerland;; f Division of Infectious Diseases, Regional Hospital Lugano, University of Geneva and University of Southern Switzerland, Lugano, Switzerland;; g Laboratory of Virology and Division of Infectious Diseases, Geneva University Hospital, University of Geneva, Geneva, Switzerland;; h Division of Infectious Diseases, Lausanne University Hospital, Lausanne, Switzerland;; i Department of Internal Medicine III with Haematology, Medical Oncology, Haemostaseology, Infectiology and Rheumatology, Oncologic Center, Paracelsus Medical University, Salzburg, Austria;; j Department of Dermatology, Medical University Vienna, Vienna, Austria;; k Institute of Hospital Hygiene and Microbiology, Styrian Hospital Corporation, The Styrian Healthcare Company, Graz, Austria;; l Molecular Virology, Department of Biomedicine–Petersplatz, University of Basel, Basel, Switzerland;; m Department of Dermatology, Kepler University Hospital, Linz, Austria;; n Faculty of Biology and Medicine, University of Lausanne, Lausanne, Switzerland;; o Department of Infectious Diseases, Bern University Hospital, University of Bern, Bern, Switzerland;; p Division of Infectious Diseases, Cantonal Hospital St Gallen, St. Gallen, Switzerland; and; q Department of Dermatology, Venereology and Allergology, Medical University of Innsbruck, Innsbruck, Austria.

**Keywords:** epidemiology, HIV, phylogenetics, transmission patterns

## Abstract

Phylogenetic analyses of 2 or more countries allow to detect differences in transmission dynamics of local HIV-1 epidemics beyond differences in demographic characteristics.

**Methods:**

A maximum-likelihood phylogenetic tree was built using *pol*-sequences of the Swiss HIV Cohort Study (SHCS) and the Austrian HIV Cohort Study (AHIVCOS), with international background sequences. Three types of phylogenetic cherries (clusters of size 2) were analyzed further: (1) domestic cherries; (2) international cherries; and (3) SHCS/AHIVCOS-cherries. Transmission group and ethnicities observed within the cherries were compared with the respective distribution expected from a random distribution of patients on the phylogeny.

**Results:**

The demographic characteristics of the AHIVCOS (included patients: 3′141) and the SHCS (included patients: 12′902) are very similar. In the AHIVCOS, 36.5% of the patients were in domestic cherries, 8.3% in international cherries, and 7.0% in SHCS/AHIVCOS cherries. Similarly, in the SHCS, 43.0% of the patients were in domestic cherries, 8.2% in international cherries, and 1.7% in SHCS/AHIVCOS cherries. Although international cherries in the SHCS were dominated by heterosexuals with men who have sex with men being underrepresented, the opposite was the case for the AHIVCOS. In both cohorts, cherries with one patient belonging to the transmission group intravenous drug user and the other one non–intravenous drug user were underrepresented.

**Conclusions:**

In both cohorts, international HIV transmission plays a major role in the local epidemics, mostly driven by men who have sex with men in the AHIVOS, and by heterosexuals in the SHCS, highlighting the importance of international collaborations to understand global HIV transmission links on the way to eliminate HIV.

## Abstract 2

Supplemental Digital Content is Available in the Text.

## INTRODUCTION

Combination antiretroviral therapy (cART) cannot cure HIV infection but has the potential to curb the HIV epidemic because individuals under successful cART are not infectious.^1–3^ In resource-rich countries, there is almost universal HIV treatment available for people living with HIV (PLWH). Nevertheless, there is still ongoing HIV transmission, driven by key populations, with around 11% decrease in the number of new HIV infections in 2010–2020 in Western and Central Europe and North America.^4^ Reasons why a stronger decline of new HIV infections has not been achieved yet are manifold, including delayed diagnosis of PLWH and ongoing national and international transmission. Transmission patterns differ between countries because of differences in population structure, policies, culture, and the level of influence by global HIV epidemics, that is, travelling and migration. It is crucial to understand the local transmission network to inform policy makers about weaknesses in the cascade of care, education, and awareness about HIV in their own country.

Phylogenetic methods have been widely used to help understanding local transmission patterns of HIV epidemics in different countries and regions.^5–11^ So far, there is no consensus in how to build a phylogeny or define transmission clusters, making it difficult to compare different studies.^12,13^ However, such a comparison might be particularly useful in the case of neighboring countries with similar HIV epidemics, for example, to quantify the impact of different public health decisions on certain HIV transmission dynamics. In the case of an active exchange and commuting between neighboring countries, a comparison of results from phylogenetic analyses might be complicated by a potential nonnegligible mutual impact.

Switzerland and Austria are 2 resource-rich neighboring countries of similar population size and culture. The characteristics of the respective HIV epidemics are comparable: The numbers of new HIV diagnoses were 425 and 421 in Switzerland, and 323 and 336 (plus 74 and 94 anonymous) in Austria, in 2018 and 2019, respectively.^14,15^ The densely sampled drug resistance sequence data base of the Swiss HIV Cohort Study (SHCS) was used in several previous projects to analyze key aspects of the HIV epidemics in Switzerland.^5,6,16–19^ So far, the drug resistance sequence data base of the Austrian HIV Cohort Study (AHIVCOS) in Austria was not used for a nation-wide phylogenetic analysis to study local transmission patterns, but subsets were used for collaborations.^20,21^

Our main aim is to compare transmission dynamics of the local epidemics of Austria and Switzerland, including a quantification of infections occurring outside the country and between these 2 countries.

## METHODS

### The Cohorts

The SHCS, launched in 1988, is a prospective, multicenter cohort study enrolling adult PLWH in Switzerland. The SHCS is a nation-wide cohort with 7 centers and represents more than 70% of people on cART in Switzerland.^22,23^ The SHCS drug resistance database contains HIV partial polymerase (*pol)* sequences from around 65% of patients across all centers. The AHIVCOS was initiated in 2001 and represents about 74% of people currently receiving cART in 9 centers in Austria,^24^ with HIV sequences available from around one-third of the patients. All patients gave informed consent for participation in the SHCS or AHIVCOS. Detailed information about patient characteristics in these 2 cohorts can be found here: SHCS^25^ and AHIVCOS.^26^

### Definitions

HIV transmission group was defined as the most likely source of HIV infection self-reported by the patient: men who have sex with men (MSM), heterosexual contacts (HET), intravenous drug use (IDU), and other or unknown transmission route. HIV subtypes for descriptive purposes were determined using the Context-based Modeling for Expeditious Typing tool for classification of HIV-1 subtypes.^27^

### Construction of the Phylogeny

We compared SHCS and AHIVCOS sequences, without prior subtyping, to all non-Swiss and non-Austrian sequences from the international Los Alamos (LA) database using *Basic Local Alignment Search Tool* (BLAST, https://blast.ncbi.nlm.nih.gov/Blast.cgi), (LA download March 2019). Non-Swiss and non-Austrian LA sequences with at least 90% identity with one of the cohort sequences were selected. All SHCS, AHIVCOS, and LA sequences were aligned against the reference genome HXB2 (accession number: K03455.1) using *Multiple Sequence Comparison by Log-Expectation* (MUSCLE). Nucleotide positions for the most common cART drug resistance mutations, based on the Stanford and International Antiviral Society USA drug resistance mutations list,^28,29^ were deleted to avoid a bias introduced by cART-driven convergent evolution. We built a maximum-likelihood phylogenetic tree using the generalized time-reversible model of nucleotide evolution and the CAT approximation for rate variation across sites of *FastTree*.^30–32^ This approach of building a phylogeny was used and validated in other SHCS projects.^18,33^

### Extraction Cherries

We extracted all monophyletic pairs (henceforth called “cherries”) with at least one patient being in the SHCS or AHIVCOS, using the tree package *Analyses of Phylogenetics and Evolution*.^34^ Only cherries with a maximal cophenetic distance of 0.045 were considered.^17,35,36^ We concentrated on 3 types of cherries: (1) *Domestic cherries* with both patients being in the same cohort, ie, AHIVCOS or SHCS, termed AHIVCOS/AHIVCOS-cherries or SHCS/SHCS-cherries; (2) *International cherries* with one patient in the AHIVCOS or SHCS and the other patient from the LA database, termed AHIVCOS/LA-cherries or SHCS/LA-cherries; and (3) *SHCS/AHIVCOS-cherries* with one patient being from the SHCS and the other patient being from the AHIVCOS.

### Sensitivity Analysis

We repeated all analyses by stepwise narrowing the cophenetic distance threshold from 0.045 to 0.015.^37^ Moreover, because the SHCS is more densely sampled as compared with the AHIVCOS, we performed simulation analyses by stepwise downsampling the SHCS sequence data set: we trimmed the original phylogenetic tree by randomly cutting up to 80% of the SHCS tree tips and extracting new sets of cherries from the trimmed trees. For each given SHCS sample proportion, the procedure was repeated 100 times, and the results were averaged.^38^

### Statistical Analysis

#### HIV Subtype Distribution

We counted the number of different subtype cherries for the different cherry types (domestic, international and SHCS/AHIVCOS-cherries).

#### Age

For domestic cherries and SHCS/AHIVCOS-cherries, we determined the age difference of the patients based on the birth year of the patient and compared the 2 cohorts using the Wilcoxon test. For LA sequences, no age information was available.

#### Transmission Group and Ethnicity

We first determined the frequencies of the traits among SHCS and AHIVCOS patients in the tree. We then analyzed the 3 types of cherries: (1) *Domestic cherries*: Based on the occurrence of traits in the tree, we calculated the frequencies of traits one would expect by randomly pairing patients of the same cohort. We call the ratio of the expected and observed pairings of traits in the SHCS/SHCS-cherries and AHIVCOS-cherries *assortativity factor (AF)*. (2) *International cherries*: We compared the frequency of traits in the tree with the frequency of traits in international cherries. The ratio of these frequencies was then used to assess whether a trait is more or less common in SHCS/LA-cherries or AHIVCOS LA-cherries than expected from the frequency of traits on the tree (3) *AHIVCOS/SHCS-cherries*: Similarly, we used the ratio of observed and expected distributions (based on the trait distribution in the whole tree) to assess whether traits are more or less common as compared with randomly pairing SHCS and AHIVCOS-patients. See Supplementary material Section S1, Supplemental Digital Content, http://links.lww.com/QAI/B829 for the detailed description of the formulas. We used *MultinomCI* of the R package *DescTool*^39^ for calculating confidence intervals of categorical variables, that is, the distribution of the traits in the cherries, and with that derived confidence intervals for the ratios and AFs.

## RESULTS

### Patient Characteristics and Number of Cherries

We included 3141 AHIVCOS and 12902 SHCS patients in the phylogenetic tree. Of the 188917 background sequences downloaded from the LA database, 7970 sequences were included in the phylogenetic tree. The majority of SHCS and AHIVCOS patients was male, of white ethnicity, and the transmission group MSM (Table 1). See Table S1 and S2, Supplemental Digital Content, http://links.lww.com/QAI/B829 for the characteristics of all patients in the cohorts as compared with patients in the phylogeny. We obtained 1148 AHIVCOS patients (36.5%) in domestic cherries and 260 (8.3%) in international cherries. Similarly, SHCS patients were predominantly in domestic cherries (5544, 43.0%) as compared with international cherries (1061, 8.2%). We obtained 220 SHCS/AHIVCOS-cherries, reflecting 1.7% of all SHCS and 7.0% of all AHIVCOS patients. We recalculated the number of different cherry types for different distance thresholds and sampling densities of the SHCS: With an SHCS sample density of 0.5, that is, randomly selecting around 50% of the SHCS sequences, we obtain a similar percentage of domestic SHCS cherries as compared with domestic AHIVCOS cherries (37.4% vs 36.4%) (see Table S3 and S4, Supplemental Digital Content, http://links.lww.com/QAI/B829). At the same time, after downsampling the SHCS to 50% of the sequences, the percentage of international SHCS cherries increases to 11.1%, compared with 9% international cherries in the AHIVCOS, pointing toward a similar fraction of international cherries in the SHCS and the AHIVCOS (see Table S5 and S6, Supplemental Digital Content, http://links.lww.com/QAI/B829). Moreover, the fraction of AHIVCOS patients in AHIVCOS/SHCS-cherries is higher as compared with SHCS patients in AHIVCOS/SHCS-cherries, even after downsampling 50% of the SHCS sequences (see Table S7 and S8, Supplemental Digital Content, http://links.lww.com/QAI/B829). Of note, the total number of AHIVCOS/SHCS-cherries, that is, 220, is higher than the number of SHCS/LA-cherries with the LA sequence from the United States, the country with most links to the SHCS (189 SHCS/LA-cherries with the LA sequence from the United States). See Table S9, Supplemental Digital Content, http://links.lww.com/QAI/B829 for the countries of origin of the LA sequences in all AHIVCOS/LA- and SHCS/LA-cherries.

**TABLE 1.** Basic Characteristic of the Study Population

Total		AHIVCOS	SHCS
		3141	12,902
Cohort center		Vienna: 1837 (58.5%)	Zürich: 4869 (37.7%)
		Linz: 487 (15.5%)	Lausanne: 1911 (14.8%)
		Graz: 354 (11.3%)	Geneva: 1762 (13.7%)
		Innsbruck: 235 (7.5%)	Bern: 1712 (13.3%)
		Salzburg: 228 (7.3%)	Basel: 1427 (11.1%)
			St Gallen: 833 (6.5%)
			Lugano: 388 (3.0%)
Sex	Male	2375 (75.6%)	9272 (71.9%)
	Female	766 (24.4%)	3630 (28.1%)
Birth year	Median (IQR)	1972 (1964–1981)	1965 (1959–1972)
Registration year	Median (IQR)	2009 (2003–2013)	2001 (1996–2009)
Sequence year	Median (IQR)	2010 (2007–2014)	2003 (1998–2009)
ART naive at sequence date		2177 (69.3%)	8879 (68.8%)
Transmission group	MSM	1361 (43.3%)	5168 (40.1%)
	Male HET	562 (17.9%)	2133 (16.5%)
	Female HET	559 (17.8%)	2491 (19.3%)
	Male IDU	335 (10.7%)	1643 (12.7%)
	Female IDU	158 (5.0%)	887 (6.9%)
	Male other	117 (3.7%)	357 (2.8%)
	Female other	49 (1.6%)	223 (1.7%)
Ethnicity	White	2541 (80.9%)	9881 (76.6%)
	Black	303 (9.6%)	1638 (12.7%)
	Hispanic	24 (0.8%)	397 (3.1%)
	Asian	80 (2.5%)	425 (3.3%)
HIV subtype	B	2031 (64.7%)	9574 (74.2%)
	CRF01_AE	189 (6.0%)	539 (4.2%)
	CRF02_AG	128 (4.1%)	508 (3.9%)
	A	268 (8.5%)	610 (4.7%)
	C	181 (5.8%)	558 (4.3%)
	F	106 (3.4%)	158 (1.2%)
	G	78 (2.5%)	300 (2.3%)
	D	31 (1.0%)	157 (1.2%)
	Other or unknown	446 (14.2%)	1545 (12.0%)

ART, antiretroviral therapy; IQR, interquartile range; IDU, intravenous drug use.

### Subtype Distribution and Age Difference

**FIGURE 1.**
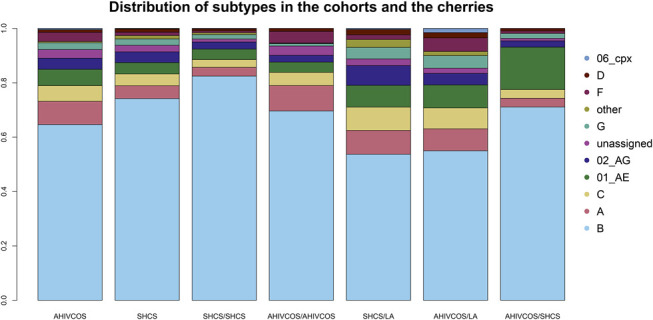
Distribution of subtype in the different types of cherries: SHCS/SHCS, AHIVCOS/AHIVCOS, SHCS/LA, AHIVCOS/LA, and AHIVCOS/SHCS.

**FIGURE 2.**
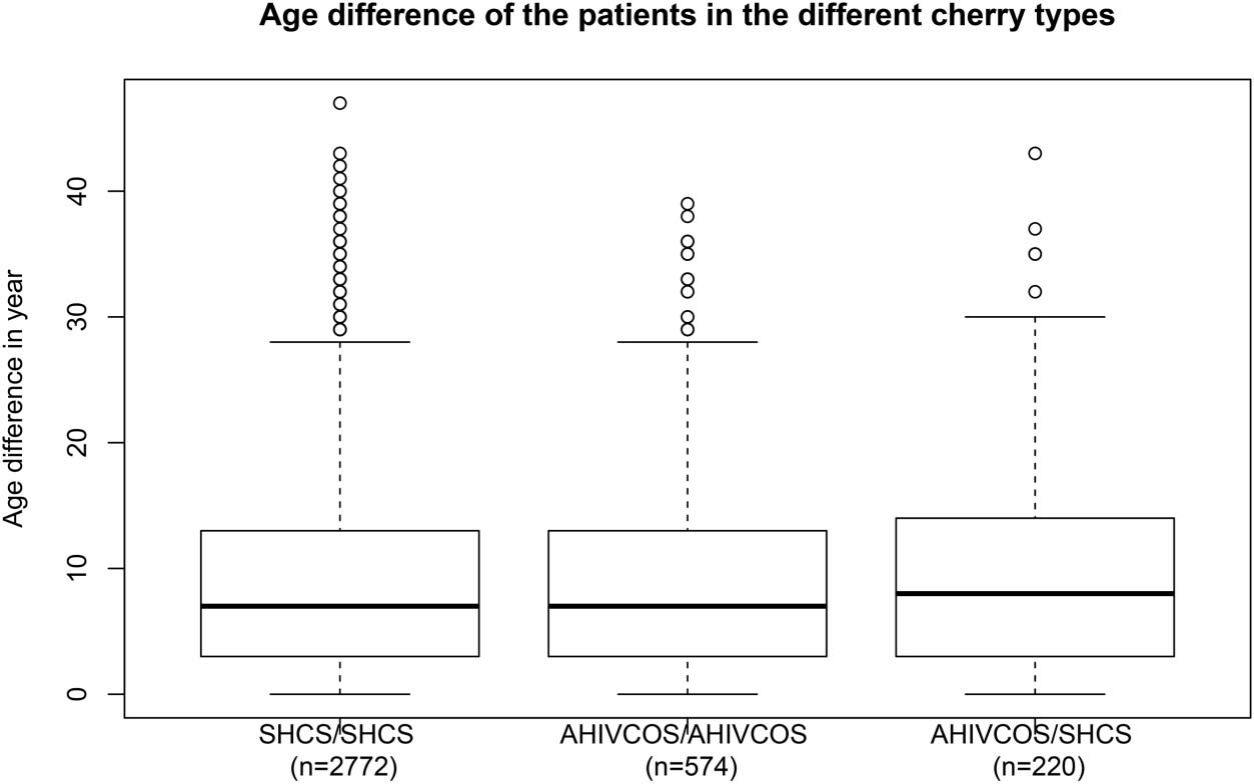
The distribution of the age difference in the different cherry types: Domestic cherries (SHCS/SHCS, AHIVCOS/AHIVCOS) and AHIVCOS/SHCS cherries

The fraction of HIV subtype B cherries was highest in SHCS/SHCS-cherries (2230, 82.5%), followed by AHIVCOS/SHCS-cherries (155%, 71.1%) and AHIVCOS/AHIVCOS-cherries (388%, 69.7%). In the case of international cherries, the subtype distribution between SHCS and AHIVCOS cherries was similar, except for subtype 02_AG and F (see Fig. 1). The fraction of subtype B cherries was lower for both cohorts (AHIVCOS/LA-cherries: 143 (55%), SHCS/LA-cherries: 570 (53.7%)) as compared with the fraction of subtype B participants in the cohorts. The median age difference in SHCS/SHCS-cherries as well as AHIVCOS/AHIVCOS-cherries was 7 years (interquartile range = 3–13 years), with no significant difference in the age difference (*P* = 0.51). The median age difference in AHIVCOS/SHCS-cherries was 8 years (interquartile range = 3–14 years), see Figure 2.

### Transmission Group and Ethnicity of Domestic Cherries

**FIGURE 3.**
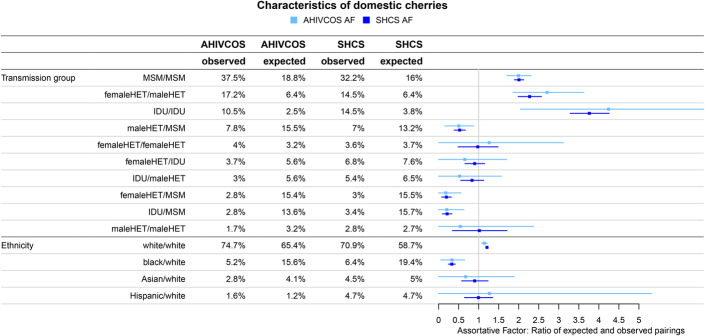
Characteristics of domestic cherries: Frequency of cherries with different patient characteristics and the AF, that is, the observed frequency divided by the expected frequency when randomly pairing cohort patients.

In both cohorts, MSM/MSM, IDU/IDU, and male HET/female HET cherries were overrepresented, that is, they were more frequent than expected by randomly pairing patients in the cohorts. This corresponds to an AF greater than one for these pairs (see Methods). IDU/IDU-cherries were most assortative (AHIVCOS AF = 4.24, SHCS AF = 3.76), followed by female HET/male HET-cherries (AHIVCOS AF = 2.71, SHCS AF = 2.27) and MSM/MSM-cherries (AHVICOS AF = 2.00, SHCS AF = 2.01) (see Fig. 3 for all traits). Of note, IDU/non-IDU cherries were more common in the SHCS (AF = 0.52) as compared with the AHIVCOS (AF = 0.38). The assortativity regarding white ethnicity was similar in both cohorts (AHIVCOS AF = 1.14, SHCS AF = 1.21). All differences between SHCS and AHIVCOS domestic cherries were stable with respect to downsampling and varying the cophenetic distance threshold: In the case of IDU/IDU-cherries, the AF ranged between 3.5 and 5.2, indicating a clear overrepresentation of this cherry type and still higher as compared with MSM/MSM-cherries (range 2.0–2.6) (Figures S3 and S4, Supplemental Digital Content, http://links.lww.com/QAI/B829). The situation was less clear in the case of female HET/female HET-cherries with range 0.7–1.4, but the AF was clearly higher in the SHCS regardless of the distance threshold and SHCS sample density (Figure S5, Supplemental Digital Content, http://links.lww.com/QAI/B829). The AF concerning ethnicity was very stable for both cohorts (Figure S6, Supplemental Digital Content, http://links.lww.com/QAI/B829).

### Characteristics of International Cherries

**FIGURE 4.**
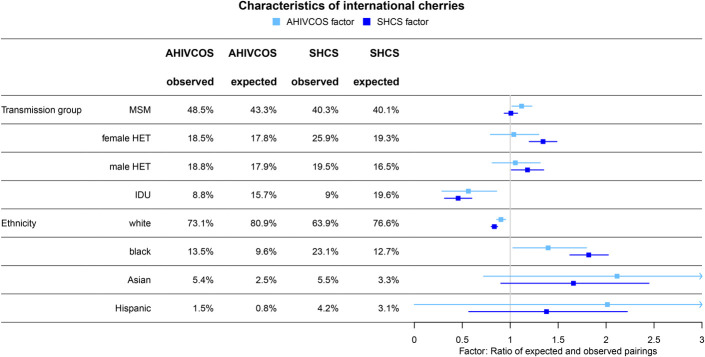
Characteristics of international cherries: Frequency of cherries with different patient characteristics and the factor: observed frequency divided by the frequency in the cohort (expected frequency).

In the SHCS, international cherries were dominated by HET (male HET: 19.5%, ratio = 1.18, female HET: 25.9%, ratio = 1.34), whereas MSM were not overrepresented (40.3%, ratio = 1.01). In contrast, in the AHIVCOS, international cherries were dominated by MSM (48.5%, ratio = 1.12), whereas HET were only slightly overrepresented (male HET: 18.8%, ratio = 1.05, female HET: 18.5%, ratio = 1.04). In both cohorts, IDU were underrepresented in international cherries, in the SHCS even more as compared with the AHIVCOS (AHIVCOS ratio: 0.56, SHCS ratio: 0.46). Similarly, in both cohorts, patients of white ethnicity were less present in international cherries as would be expected from the cohort distribution (AHIVCOS ratio: 0.90; SHCS ratio: 0.83). Contrariwise, patients of black and Asian ethnicity were more frequent in international cherries as compared with the frequency in the cohort (see Fig. 4). These results were stable in our sensitivity analysis, with the ratio for IDU being below 1 (under-represented) and for MSM above 1 (over-represented), see Figure S7 and S8, Supplemental Digital Content, http://links.lww.com/QAI/B829. In the case of ethnicity, however, the ratio was approaching 1 for a very low distance, most likely because of the low sample size for non-white patients in international cherries of low distance (see Figure S9, Supplemental Digital Content, http://links.lww.com/QAI/B829).

In the AHIVCOS, the most frequent link in international cherries were sequences from Germany (43%, 11.7%), which was only the case in 84 (6.1%) of the SHCS sequences, with the United States having most international links to the SHCS (189%, 13.7%). See Table S7, Supplemental Digital Content, http://links.lww.com/QAI/B829 for all countries in SHCS/LA- and AHIVCOS/LA-cherries (see Table S9, Supplemental Digital Content, http://links.lww.com/QAI/B829).

### SHCS/AHIVCOS-Cherries

Of the 220 SHCS/AHIVCOS-cherries, 57 (25.9%) were MSM/MSM-cherries, followed by 21 (9.5%) male HET/female HET-cherries and 18 (8.1%) male HET/male HET-cherries. The only transmission group combination that was significantly overrepresented, as compared with the distribution expected by randomly pairing SHCS and AHIVCOS patients, were MSM/MSM-cherries (ratio = 1.49). Table 2 shows all transmission group combinations and ethnicities in SHCS/AHIVCOS-cherries.

**TABLE 2.** Transmission Group and Ethnicities in AHIVCOS/SHCS-Cherries: The Factor of the Expected and Observed Frequency of the Different Cherries and Confidence Interval

SHCS/AHIVCOS	MSM	Male HET	Female HET	Male IDU	Female IDU	Male Other	Female Other
MSM	1.49 [1.18, 1.83]	0.32 [0, 1.12]	0.33 [0, 1.02]	0.49 [0, 1.54]	0.15 [0, 2.09]	1.52 [0, 6.33]	
Male HET	0.7 [0, 1.5]	2.77 [0.92, 4.72]	1.18 [0, 2.86]	0.8 [0, 3.33]	1.11 [0, 5.8]		2.94 [0, 21.6]
Female HET	0.45 [0, 1.26]	1.85 [0, 3.82]	1.46 [0, 3.13]	1.2 [0, 3.75]	0.37 [0, 5.09]	0.92 [0, 12.64]	1.48 [0, 20.24]
Male IDU	0.64 [0, 1.99]	1.29 [0, 4.56]	1.99 [0, 4.79]	1.67 [0, 5.92]	1.24 [0, 9.11]	1.54 [0, 21.1]	
Female IDU	0.45 [0, 3.32]		0.94 [0, 6.88]	4.26 [0, 13.27]	3.94 [0, 20.63]		
Male other	1.22 [0, 5.09]	0.74 [0, 10.11]	1.26 [0, 9.29]	0.96 [0, 13.13]			
Female other		3.52 [0, 25.9]	3.02 [0, 22.18]	2.29 [0, 31.34]			16.86 [0, 230.92]

SHCS/AHIVCOS	White	Black	Asian	Hispanic	Other
White	1.09 [1.01, 1.19]	0.35 [0, 0.94]	1.54 [0, 3.79]	0.73 [0, 3.15]	1.42 [0, 3.13]
Black	0.31 [0, 1.12]	2.23 [0, 7.14]			1.08 [0, 15.43]
Asian	1.86 [0, 4.95]		21.67 [0, 93.37]		
Hispanic	0.78 [0, 11.06]				
Other	1.06 [0, 2.34]		6.74 [0, 36.46]		

## DISCUSSION

Building a phylogenetic tree, including Austrian, Swiss, and international HIV-1 sequences, revealed interesting insights into national and international transmission patterns. In both cohorts, the SHCS and AHIVCOS, around 50% of all patients were in a phylogenetic cherry. In the AHIVCOS, 30% of the patients in a cherry had a link to a non-Austrian sequence (16% international LA database and 13.5% Switzerland). Similarly, a significant amount of SHCS patients had links to non-Swiss sequences (15.5% international LA sequences and 3.2% Austria). Given that the LA background sequence database is less representative of the global HIV-1 epidemic as compared with the 2 local cohorts, we can assume that the fraction of international, that is, non-Austrian and non-Swiss, sequences is underrepresented. This means that in both countries, international links have a major impact on the local HIV-1 epidemics. This highlights the importance of transnational collaboration to understand the dynamics of the on-going HIV-1 epidemics. By combining the sequence databases of the Austrian and Swiss cohorts, we were able to compare transmission in the 2 local epidemics. Regarding links to the LA database, we could identify differences in transmission groups in the 2 countries: Although in Austria, international links are dominated by MSM, in Switzerland, international links are overrepresented by HET. This indicates differences in international HIV-1 transmission sources between Austria and Switzerland. The interpretation of our results is that in Austria, the HIV-1 epidemic among MSM is more influenced by international transmission, that is, MSM being infected by their partner abroad, as compared with Switzerland. Of note, because we do not distinguish between nationalities in this project, our findings only reflect the amount of transmission events between patients registered in the local cohorts (SHCS or AHIVCOS) and patients somewhere else, but it does not tell us anything about the role of immigrants in the respective countries, as immigrants are part of the local cohorts too and hence count as domestic transmission. In Switzerland, it was shown before that the HIV-1 epidemic among HET is not self-sustained, indicating the major impact of international transmission and domestic transmission of other transmission groups in the case of HET.^18^ In both cohorts, few international links were found among IDU, indicating that HIV-1 transmission among IDU predominantly occurred within local transmission networks, in Switzerland even more than in Austria. This mostly domestic transmission dynamics of HIV-1 among IDU might have helped the successful prevention and virtual eradication of HIV transmission among IDU in Switzerland and presumably also in Austria.^19^

Combining Austrian and Swiss sequences into one phylogenetic tree allows to study and compare characteristics of the local epidemics. In both cohorts, cherries of the expected HIV-1 transmission group combinations, that is, MSM/MSM, IDU/IDU, and male HET/female HET were most assortative, in the AHIVCOS even more as compared with the SHCS. In the SHCS, IDU/non-IDU, that is, IDU/MSM, IDU/male HET, and IDU/female HET, were more frequent as compared with the AHIVCOS. This suggests that the overspill of the HIV epidemic among IDU to other transmission groups was larger in Switzerland as compared with Austria. Interestingly, the AF of female HET/female HET pairs, the transmission group combination with a very small HIV transmission probability, was above 1 in both cohorts. Although not statistically significant, this is an indication of unsampled male HET in both cohorts, in the SHCS even more than in the AHIVCOS. In Switzerland and in Austria, HIV testing is done routinely in pregnant women, and hence, female HET have a higher chance of being diagnosed. In addition, there might be more reluctance toward HIV testing in male HET as compared with female HET in general, as was observed for other countries.^40^

Building a phylogenetic tree, extracting clusters from the tree, and analyzing properties of clusters involve numerous modelling and parameter choices. To date, there is no consensus regarding the ideal way to construct an HIV phylogeny, neither for extraction of HIV transmission clusters.^12,13^ Comparing results from different publications, such as the fraction of international transmission links or properties of local transmission clusters, is hence rarely possible. To target HIV-1 prevention, it is important to understand where previous prevention campaigns are lagging behind, also in comparison to other countries. A comparison is only possible if the same methods were used to quantify the respective problem. Using the “HIV estimates accuracy tool” provided by the European Center for Disease Control,^41,42^ estimates concerning local HIV epidemics can be made, such as estimates about the percentage of undiagnosed HIV-infected people. With this tool, it is hence possible to compare the WHO 90-90-90 goals between different countries, using the same method, differences in the sample density and missing data are taken into account. To our knowledge, no such tool is available including phylogenetic analyses. Hence, our study showcases how combined phylogenies could be used to understand, compare, and quantify transmission patterns of local epidemics.

Ragonnet-Cronin et al ^36^ performed a phylogenetic study to compare transmission patterns of the HIV epidemics in Switzerland and the United Kingdom by applying the same methods on the drug resistance database of the SHCS and the United Kingdom HIV resistance data base. They found similar characteristics of the Swiss and United Kingdom local HIV epidemics after correcting for differences in sample size. In our comparison of Switzerland and Austria, in addition to analyzing the local epidemics, we investigate properties of international links with the LA database, as well as properties of potential overlaps between the Swiss and Austrian HIV epidemic. In contrast to the study of Ragonnet-Cronin et al, our approach however necessitates the transfer of sequences from one cohort to the other cohort.

Our study has several strengths and limitations. One strength is that for showcasing the use of a combined phylogeny, we were able to include Switzerland and Austria, 2 neighboring countries of comparable size, culture, and similar basic HIV epidemics. One major limitation is inevitable to all phylogenetic analyses: throughout the construction of the tree and extraction of phylogenetic clusters, a multitude of parameters need to be chosen. In this project, we concentrated on clusters of size 2, “cherries,” based on the tree topology and an additional genetic distance criterion. On the one hand, this simplification disregards a lot of sequences potentially closely clustered with these cherries; on the other hand, it provides an intuitive interpretation of the transmission patterns through the proposed assortative factor. However, previous work has shown that transmission characteristics of cherries are a good proxy of characteristics of larger clusters.^43^

Extensive sensitivity analyses were performed to understand the impact of sampling density and distance threshold for the cherry definition on our results and performed numerous simulations (Section S6, Supplemental Digital Content, http://links.lww.com/QAI/B829). In addition, we performed a sensitivity analysis by rebuilding the phylogenetic tree with subtype B sequences only. The distribution of characteristics is very similar, indicating robustness of our results (see Section S7.1, Supplemental Digital Content, http://links.lww.com/QAI/B829). Similarly, we rebuilt the phylogenies of Austrian and Swiss sequences separately, again with robust results (see Section S7.2, Supplemental Digital Content, http://links.lww.com/QAI/B829). Furthermore, the SHCS has sequenced a large number of samples retrospectively for research purposes. As a sensitivity analysis, we rebuilt the phylogenetic tree with retrospectively sequenced samples removed and obtain similar results as presented in the main analysis (see Section S8, Supplemental Digital Content, http://links.lww.com/QAI/B829). Another limitation is that sequences included in the LA database might be biased and do not reflect the trait distribution of the respective countries. Given the higher median genetic distance in international cherries as compared with domestic cherries, we conclude that the international cherries observed in our project most likely do not reflect direct transmission events but rather 2 sequences on a transmission chain with few intermediate transmission events. Hence, the country of origin of the LA sequences might not reflect the countries of the direct transmission events. One of the main characteristics we study is the HIV transmission route, which is self-reported by the patients, and potentially underestimates the assortativeness of patients from the same transmission group.

In summary, the local epidemics of Austria and Switzerland are of remarkable similarity, with only minor differences observed in transmission patterns. In both cohorts, international transmission links play a major role, mainly driven by MSM in Austria and HET in Switzerland. This underlines the importance of international collaborations to understand the links between HIV epidemics in different areas on the way to eliminate HIV. The overrepresentation of female HET cherries indicates missing HIV diagnoses of male HET in both cohorts, calling for tailored HIV testing strategies among male HET. Moreover, the underrepresentation of IDU in international cherries in both cohorts highlight the success of the virtual elimination of HIV transmission among IDU.

## ETHICAL STATEMENT

AHIVCOS: Approval for this study was obtained from the local ethical committees of all participating centers: ethics committee of the Vienna Medical University (No. 898/2010), of the Salzburg Federal Government (No. 1159/2010), of the Graz Medical University (No. 21–431/2010), of the Innsbruck Medical University (No 283/4.4/2009), of the Upper Austria Federal Government (No C-3-10/2010), and of the Carynthian Federal State (No A-13-11/2011). Written informed consent was given by the patients for their information to be stored in the hospital database and used for research.

SHCS: The SHCS was approved by the local ethical committees of the participating centers: Ethikkommission beider Basel (“Die Ethikkommission beider Basel hat die Dokumente zur Studie zustimmend zur Kenntnis genommen und genehmigt.”); Kantonale Ethikkommission Bern (21/88); Comité départemental d'éthique des spécialités médicales et de médecine communautaire et de premier recours, Hôpitaux Universitaires de Genève (01–142); Commission cantonale d'éthique de la recherche sur l'être humain, Canton de Vaud (131/01); Comitato etico cantonale, Repubblica e Cantone Ticino (CE 813); Ethikkommission des Kantons St. Gallen (EKSG 12/003); Kantonale Ethikkommission Zürich (KEK-ZH-NR: EK-793), and written informed consent was obtained from all participants.

## DATA AVAILABILITY STATEMENT

The individual level data sets generated or analyzed during the current study do not fulfill the requirement for open data access: (1) The SHCS informed consent states that sharing data outside the SHCS network is only permitted for specific studies on HIV infection and its complications and to researchers who have signed an agreement detailing the use of the data and biological samples; and (2) the data is too dense and comprehensive to preserve patient privacy in persons living with HIV. According to the Swiss law, data cannot be shared if data subjects have not agreed or data are too sensitive to share. Investigators with a request for selected data should send a proposal to the respective SHCS address (www.shcs.ch/contact). The provision of data will be considered by the Scientific Board of the SHCS and the study team and is subject to Swiss legal and ethical regulations, and it is outlined in a material and data transfer agreement.
